# Supplementary material for: Peer-led interventions: Exploring the peer group leader experience of delivering Sauti ya Vijana, a group-based mental health intervention for youth living with HIV in Tanzania
Source: PLOS Ment Health. 2026 Jan 21;3(1):e0000512. doi: 10.1371/journal.pmen.0000512 (PMC12822943; doi:10.1371/journal.pmen.0000512)
Supplement: S1 Text — (DOCX) [file pmen.0000512.s001.docx]

Table 1: *Description of the 25 PGLs*

| Variable | | N | | Percentage | |
| --- | --- | --- | --- | --- | --- |
| Age (at time of interview in 2023)* | | | | | |
| Mean (range) | | 26 (23-31) | |  | |
| Gender | | | | | |
| Female | | 12 | | 48% | |
| Male | | 13 | | 52% | |
| Marital Status | | | | | |
| Single | | 6 | | 24% | |
| In a relationship | | 13 | | 52% | |
| Married | | 6 | | 24% | |
| Has Children | | | | | |
| Yes | | 16 | | 64% | |
| Secondary Job | | | | | |
| Yes | | 11 | | 44% | |
| Education Level | | | | | |
| Primary education | | 3 | | 12% | |
| Secondary education (Form 1-4) | | 14 | | 56% | |
| Higher education | | 8 | | 32% | |
| Type of Higher Education | | | | | |
| College/Vocational | | 5 | | 20% | |
| University | | 3 | | 12% | |
| Religion | | | | | |
| Christian | | 20 | | 80% | |
| Muslim | | 5 | | 20% | |

Note. *N = 25. PGLs were hired in 2021. Interviews took place approximately 2 years later.*
