# Supplementary material for: Peer-led interventions: Exploring the peer group leader experience of delivering Sauti ya Vijana, a group-based mental health intervention for youth living with HIV in Tanzania
Source: PLOS Ment Health. 2026 Jan 21;3(1):e0000512. doi: 10.1371/journal.pmen.0000512 (PMC12822943; doi:10.1371/journal.pmen.0000512)
Supplement: S2 Text — (DOCX) [file pmen.0000512.s002.docx]

In-Depth Interviews Question Guide

| 1. Interviewer name |  |
| --- | --- |
| 1. Participant ID# |  |
| 1. Interview date (dd/mm/yyyy) | \|___\|___\|/\|___\|___\|/\|___\|___\|___\|___\| |
| 1. Participant Age |  |
| 1. Participant Gender |  |
| 1. Participant agrees to digitally record interview | Yes  No |
| 1. Time interview began (hh:mm) | \|___\|___ \| : \|___\|___\| am/pm |
| 1. Time interview ended (hh:mm) | \|___\|___\| : \|___\|___\| am/pm |

Step 1: Complete Q1—3 above before starting the interview.

Step 2: Introduce yourself at the beginning of the interview.

Step 3: Thank participant for taking part in the interview.

Step 4: Read Section 1: Information about the study to the participant.

Step 5: Ask for the participant’s permission to record the interview. Tick appropriate box in Q6 above.

Step 6: Turn on audio recorder if permitted. Document time interview begins in Q7 above.

Step 7: Conduct interview.

Step 8: Thank the participant at the end of the interview. Ask if has any questions.

Step 9: Document time interview ended in Q8 above.

Step 10: Provide reimbursement and document appropriately.

Section 1: Information about the study

The goal of this interview is to learn about your experience as a group leader in the SYV program and to find out if there are ways to improve the program in the future.

There are no right or wrong answers to the questions I’ll ask, only opinions. Please feel free to share your candid thoughts. You are the expert here, and there is no one else we can ask to get the unique information that you can tell me about your beliefs and experiences.

If you agree, I would like to audio record the interview because I want to make sure I don’t miss any of your comments. If you don’t want the interview audio recorded, I will take detailed notes during the interview instead. The recording will eventually be destroyed after we publish the study’s findings.

Please know that participating in this interview is voluntary. You can choose not to answer a question or you can stop participating at any time.

You will receive 7,500 TSH for taking part in the interview. The interview will take about an hour to an hour and a half.

Do you have any questions for me so far about the interview?

*[If yes, answer the participant’s questions.]*

Are you okay with our conversation being audio recorded?

*[If yes, begin audio recording now.]*

*[If no]* That’s okay, I’ll take detailed notes as we talk.

OK -- Let’s get started!

Section 2: Identify

*Interviewer script: I’d like to start by learning a little about you, how you view yourself, and your goals for the future.*

1. *[Individual: Other personal attributes]* To start, please tell me about yourself?
   1. How do you think other people would describe you?
2. *[Individual: Other personal attributes]* When you encounter a problem, what do you normally do?
3. *[Individual: Other personal attributes]* Who, if anyone, do you reach out to if you need support or advice in your life?
   1. Why do you reach out to that person?
   2. What type of support or advice does that person usually provide?
4. *[Individual: Other personal attributes]* What do you want your life to be like 5 years from now? *[PROBE: about family, career, relationships]*

Section 3: Motivations

*Interviewer script: Now I’d like to ask you about your decision to become a Group Leader.*

1. Do you work at the clinic outside of your role as a group leader in the SYV program?
   1. [IF YES] What is your role in the clinic?
   2. What are your responsibilities in that role?
   3. How long have you served in that role?
2. *[Individual: Other personal attributes]* Why did you decide to become a group leader?
   1. What were the main reasons you wanted to become involved?
3. *[Inner: Readiness for implementation]* What steps did you do to become a group leader, such as being hired and trained?
4. *[Individual: Knowledge & beliefs about the intervention]* What did you know about the intervention before becoming a group leader?
5. *[Individual: Knowledge & beliefs about the intervention]* When you first learned of the intervention, what did you think?
   1. How have these impressions changed, if at all, since becoming a group leader?

Section 4: Role expectations

1. *[Inner: Readiness for implementation]* What concerns do you have, if any, about being a group leader?
2. *[Inner: Implementation climate]* What have been your goals as a group leader?
3. *[Individual: Self-efficacy]* Do you expect the workload as a group leader to be difficult or easy to manage?
   1. What makes you feel that way?
4. *[Individual: Knowledge & beliefs about the intervention]* What do you expect will be the primary things that you do as a group leader?

Section 5: Experience as a group leader

*Interviewer script: Let’s now talk about your experience as a Group Leader.*

1. *[Individual: Knowledge & beliefs about the intervention]* How would you describe your group leader role to others?
2. *[Individual: Other personal attributes]* How long do you plan on being a group leader?
   1. Why do you feel like you want to remain a group leader for that long?
3. *[Individual: Other personal attributes]* What has been your personal experience as a group leader so far?
   1. What does it mean to you to be a group leader?
   2. What do you think are the desired qualities of a group leader?
4. What do you enjoy the most about being a group leader?
   1. What do you like the least about being a group leader?
5. *[Inner: Networks & communications]* What are your relationships like with other group leaders?
6. How has being a group leader affected your life?
   1. How has it affected your relationships outside of work?
7. *[Inner: Implementation climate]* What kinds of incentives are there for group leaders to help ensure that the implementation of the SYV program is successful? *[Probe about both financial and non-financial incentives.]*
   1. In your opinion, are these incentives enough?
      1. What makes you feel that way?
   2. What other types of incentives would encourage group leaders to ensure the program is successful?
8. *[Inner: Readiness for implementation]* What do you think you need to be best supported as a group leader?
   1. Are there areas where you want more support?

*Interviewer script: Now let’s talk about the training you received to be a Group Leader.*

1. *[Inner: Readiness for implementation]* How did trainings prepare you for you your role?
   1. What aspects of the training did you find most helpful?
   2. How could training be improved?
2. *[Inner: Readiness for implementation]* At the start of being a group leader, did you feel prepared to take on the responsibilities associated with being a group leader?
   1. How has this changed, if at all?
3. *[Individual: Self-efficacy]* If participants have questions, how confident do you feel you will be to answer them?
   1. *[If not very confident]* What would make you feel more confident?
4. *[Inner: Networks & communications]* How did you find the supervision meetings?
   1. What would make supervision more useful?
   2. Did you feel you were able to ask questions?
      1. What makes you feel that way?
   3. How comfortable do you feel you will be to honestly report challenges you experience during sessions?

Section 6: Impact of intervention on participants

*Interviewer script: Now, I’d like to hear from you your thoughts about the affect the SYV program may have on young people living with HIV.*

1. *[Individual: Knowledge & beliefs about the intervention]* In your own words, what is the purpose of the intervention?
   1. What can you tell me about the structure and content of the intervention?
2. *[Inner: Implementation climate]* What do you think are the expectations of the young people with whom you work regarding being in the SYV intervention study?
   1. Do you feel like you and the program meets those expectations?
      1. Why or why not?
3. *[Inner: Tension for Change]* How do you think the intervention can better reach adolescents and young people who would benefit from it?

Section 7: Sustainability

*Interviewer script: Let’s now talk about how the program and lesson learned in the program can be sustained.*

1. What does sustainability mean to you?
   1. What do you think are important factors in sustaining an intervention like SYV?
2. *[Inner: Structural characteristics]* How would this intervention need to change in your opinion to be implemented at other clinics in your country?

Section 8: Recommendations

*Interviewer script: To wrap up, I’d like to hear about how we could potentially improve the SYV program.*

1. *[Inner: Structural characteristics]* How could the intervention be changed to best support youth participants?
   1. *[Acknowledge any changes mentioned earlier in the interview and probe further by asking…]* How else could the intervention be changed to better support Group Leaders?
   2. Is there anything else about the intervention you would change?
      1. *[If yes]* What about the intervention would you change?
      2. What makes you feel that way?

Section 9: Interview Closing

We are nearing the end of our conversation today.

1. Before we end, is there any other information you’d like to share about your experience as a Group Leader in the SYV program?

I want to sincerely thank you for your time and for the helpful information that you provided.

Section 10: Surveys

*Interviewer script:* I would like to finish our conversation today by asking you a few final survey questions.

*[Ask each of the survey questions and mark the participants’ response in the table below. Do NOT turn off the recorder yet (if audio recording).]*

Coping Self-Efficacy Scale

*Interviewer script:* I’ll now be asking you some questions about when things aren’t going well for you, or when you’re having problems. I would like to know how confident or certain you are that you can do the following things. As you think of your answer, please let me know how confident or certain you are using the following rating scale: Cannot do at all, Moderately certain can do, Certain can do.

| Q1. Sort out what can be changed, and what cannot be changed | Cannot do at all = 0  Moderately certain can do = 1  Certain can do = 2 |
| --- | --- |
| Q2. Get emotional support from friends and family | Cannot do at all = 0  Moderately certain can do = 1  Certain can do = 2 |
| Q3. Find solutions to your most difficult problems | Cannot do at all = 0  Moderately certain can do = 1  Certain can do = 2 |
| Q4. Break an upsetting problem down into smaller parts | Cannot do at all = 0  Moderately certain can do = 1  Certain can do = 2 |
| Q5. Leave options open when things get stressful | Cannot do at all = 0  Moderately certain can do = 1  Certain can do = 2 |
| Q6. Make a plan of action and follow it when confronted with a problem | Cannot do at all = 0  Moderately certain can do = 1  Certain can do = 2 |
| Q7. Take your mind off unpleasant thoughts | Cannot do at all = 0  Moderately certain can do = 1  Certain can do = 2 |
| Q8. Keep from feeling sad | Cannot do at all = 0  Moderately certain can do = 1  Certain can do = 2 |
| Q9. Stop yourself from being upset by unpleasant thoughts | Cannot do at all = 0  Moderately certain can do = 1  Certain can do = 2 |
| Q10. Make new friends | Cannot do at all = 0  Moderately certain can do = 1  Certain can do = 2 |
| Q11. Get friends to help you with the things you need | Cannot do at all = 0  Moderately certain can do = 1  Certain can do = 2 |
| Q12. Make unpleasant thoughts go away | Cannot do at all = 0  Moderately certain can do = 1  Certain can do = 2 |
| Q13. Think about one part of the problem at a time | Cannot do at all = 0  Moderately certain can do = 1  Certain can do = 2 |

Rosenberg Self Esteem Scale

*Interviewer script:* I’ll now be asking you some questions about your feelings. For each statement, please let me know if you strongly agree, agree, disagree, strongly disagree, or feel neutral.

| Q1. I feel that I am a person of worth, at least on an equal plane with others | Strongly Disagree = 1  Disagree = 2  Agree = 3  Strongly Agree = 4 |
| --- | --- |
| Q2. I feel that I have a number of good qualities | Strongly Disagree = 1  Disagree = 2  Agree = 3  Strongly Agree = 4 |
| Q3. All in all, I am inclined to feel I am a failure | Strongly Disagree = 1  Disagree = 2  Agree = 3  Strongly Agree = 4 |
| Q4. I am able to do things as well as most other people | Strongly Disagree = 1  Disagree = 2  Agree = 3  Strongly Agree = 4 |
| Q5. I certainly feel useless at times | Strongly Disagree = 1  Disagree = 2  Agree = 3  Strongly Agree = 4 |
| Q6. On the whole, I am satisfied with myself. | Strongly Disagree = 1  Disagree = 2  Agree = 3  Strongly Agree = 4 |
| Q7. I wish I could have more respect for myself | Strongly Disagree = 1  Disagree = 2  Agree = 3  Strongly Agree = 4 |
| Q8. I take a positive attitude toward myself. | Strongly Disagree = 1  Disagree = 2  Agree = 3  Strongly Agree = 4 |
| Q9. At times I think I am no good at all | Strongly Disagree = 1  Disagree = 2  Agree = 3  Strongly Agree = 4 |
| Q10. I feel I do not have much to be proud of | Strongly Disagree = 1  Disagree = 2  Agree = 3  Strongly Agree = 4 |

*Interviewer script:* Thank you! That is the last of my questions for you today. This has been very helpful.

[TURN OFF THE RECORDER]
