# Supplementary material for: Peer-led interventions: Exploring the peer group leader experience of delivering Sauti ya Vijana, a group-based mental health intervention for youth living with HIV in Tanzania
Source: PLOS Ment Health. 2026 Jan 21;3(1):e0000512. doi: 10.1371/journal.pmen.0000512 (PMC12822943; doi:10.1371/journal.pmen.0000512)
Supplement: S1 Codebook — (DOCX) [file pmen.0000512.s003.docx]

# Codebook for In-depth Interviews

| NODE NAME | DESCRIPTION |
| --- | --- |
| 1. Identity/Demographics | Q1. To start, please tell me about yourself?  a. How do you think other people would describe you? *** Additionally can highlight summary table at the beginning of each transcript (Q1) |
| 2-3. Support Network and Problem Solving | Q2. When you encounter a problem, what do you normally do?  Q3. Who, if anyone, do you reach out to if you need support or advice in your life? a. Why do you reach out to that person? b. What type of support or advice does that person usually provide? |
| 4. Future Goals | Q4. What do you want your life to be like 5 years from now? |
| 5. Outside Work | Q5. Do you work at the clinic outside of your role as a group leader in the SYV program? a. [IF YES] What is your role in the clinic? b. What are your responsibilities in that role? c. How long have you served in that role? |
| 6. Reason for Involvement | Q6. Why did you decide to become a group leader? a. What were the main reasons you wanted to become involved? |
| 7. Hiring and Training Process | Q7. What steps did you do to become a group leader, such as being hired and trained? |
| 8-9. Beliefs about Intervention | Q8. What did you know about the intervention before becoming a group leader?  Q9. When you first learned of the intervention, what did you think? a. How have these impressions changed, if at all, since becoming a group leader? |
| 10. Leadership Concerns | Q10. What concerns do you have, if any, about being a group leader? |
| 11. Leadership Goals | Q11. What have been your goals as a group leader? |
| 12. Perceived Workload and Management | Q12. Do you expect the workload as a group leader to be difficult or easy to manage? a. What makes you feel that way? |
| 13. Key Responsibilities | Q13. What do you expect will be the primary things that you do as a group leader? |
| 14. Description of role | Q14. How would you describe your group leader role to others? |
| 15. Duration of group leader role | Q15. How long do you plan on being a group leader? a. Why do you feel like you want to remain a group leader for that long? |
| 16. Personal Experience and Desired Qualities of a Group Leader | Q16. What has been your personal experience as a group leader so far?a. What does it mean to you to be a group leader?b. What do you think are the desired qualities of a group leader? |
| 17. Enjoyable and Challenging Aspects | Q17. What do you enjoy the most about being a group leader? a. What do you like the least about being a group leader? |
| 18. Relationship with Other Group Leaders | Q18. What are your relationships like with other group leaders? |
| 19. Personal Impact of Group Leadership | Q19. How has being a group leader affected your life? a. How has it affected your relationships outside of work? |
| 20-21. Incentives and Support | Q20 What kinds of incentives are there for group leaders to help ensure that the implementation of the SYV program is successful? [Probe about both financial and non-financial incentives.] a. In your opinion, are these incentives enough?  i. What makes you feel that way? b. What other types of incentives would encourage group leaders to ensure the program is successful?  Q21. What do you think you need to be best supported as a group leader?  a. Are there areas where you want more support? |
| 22-24. Preparation and Confidence | Q22. How did trainings prepare you for you your role? a. What aspects of the training did you find most helpful? b. How could training be improved? Q23. At the start of being a group leader, did you feel prepared to take on the responsibilities associated with being a group leader? a. How has this changed, if at all? Q24. If participants have questions, how confident do you feel you will be to answer them? a. [IF NOT VERY CONFIDENT] What would make you feel more confident? |
| 25. Perceptions of Supervision Meetings | Q25. How did you find the supervision meetings? a. What would make supervision more useful? b. Did you feel you were able to ask questions? i. What makes you feel that way? c. How comfortable do you feel you will be to honestly report challenges you experience during sessions? |
| 26. Purpose of Intervention | Q26. In your own words, what is the purpose of the intervention? a. What can you tell me about the structure and content of the intervention? |
| 27. Alignment with Youth's Expectations | Q27. What do you think are the expectations of the young people with whom you work regarding being in the SYV intervention study? a. Do you feel like you and the program meets those expectations?  i. Why or why not? |
| 28-30. Sustainability and Reach | Q28. How do you think the intervention can better reach adolescents and young people who would benefit from it? Q29. What does sustainability mean to you?   1. What do you think are important factors in sustaining an intervention like SYV?   Q30. How would this intervention need to change in your opinion to be implemented at other clinics in your country? |
| 31. Recommended Changes to Intervention | Q31. How could the intervention be changed to best support youth participants? a. How else could the intervention be changed to better support Group Leaders? b. Is there anything else about the intervention you would change? i. [IF YES] What about the intervention would you change? ii. What makes you feel that way? |
| 32. Wrap Up | Q32. Before we end, is there any other information you’d like to share about your experience as a Group Leader in the SYV program? |
| CONTENT CODES | *Always double code with structural code in transcript* |
| Economic Issues | Anytime economic issues were addressed in the interview. |
