# Supplementary material for: Peer-led interventions: Exploring the peer group leader experience of delivering Sauti ya Vijana, a group-based mental health intervention for youth living with HIV in Tanzania
Source: PLOS Ment Health. 2026 Jan 21;3(1):e0000512. doi: 10.1371/journal.pmen.0000512 (PMC12822943; doi:10.1371/journal.pmen.0000512)
Supplement: S1 Checklist — (DOCX) [file pmen.0000512.s004.docx]

**COREQ (COnsolidated criteria for REporting Qualitative research) Checklist**

A checklist of items that should be included in reports of qualitative research. You must report the page number in your manuscript where you consider each of the items listed in this checklist. If you have not included this information, either revise your manuscript accordingly before submitting or note N/A.

| **Topic** | **Item No.** | **Guide Questions/Description** | **Reported on Page No.** | **Notes** |
| --- | --- | --- | --- | --- |
| **Domain 1: Research team and reflexivity** | | |  |  |
| *Personal characteristics* | | |  |  |
| Interviewer/facilitator | 1 | Which author/s conducted the interview or focus group? | NA | Interviewers were not apart of the SYV intervention, they were externally hired. |
| Credentials | 2 | What were the researcher’s credentials? E.g. PhD, MD |  | Put Initials of each research assistant & Education credentials  F.N. (Post Graduate Diploma in Community Development- PGDCD)  J.M. (Masters Sociology)  N.A. (BA Sociology)  L.S. (BA Counseling psychology )  L.A.N. (Masters of Science in clinical psychology) |
| Occupation | 3 | What was their occupation at the time of the study? |  | Qualitative Research assistants |
| Gender | 4 | Was the researcher male or female? |  | 5 women completed the interviews. |
| Experience and training | 5 | What experience or training did the researcher have? |  | All interviewers had qualitative research experience. 2 days of training with all of the interviewers,. |
| *Relationship with participants* | | |  |  |
| Relationship established | 6 | Was a relationship established prior to study commencement? |  | None of the interviewers knew the participants prior to the study. |
| Participant knowledge of the interviewer | 7 | What did the participants know about the researcher? e.g. personal goals, reasons for doing the research |  | Participants did not know the research assistants. |
| Interviewer characteristics | 8 | What characteristics were reported about the interviewer/facilitator? e.g. Bias, assumptions, reasons and interests in the research topic |  | The interviewers were all Tanzanian. There was no bias present due to them not knowing the participants beforehand. |
| **Domain 2: Study design** | | |  |  |
| *Theoretical framework* | | |  |  |
| Methodological orientation and Theory | 9 | What methodological orientation was stated to underpin the study? e.g. grounded theory, discourse analysis, ethnography, phenomenology, content analysis |  | Interview guide was informed by the Consolidated Framework for Implementation Research (CFIR) and Thematic Analysis was conducted |
| *Participant selection* | | |  |  |
| Sampling | 10 | How were participants selected? e.g. purposive, convenience, consecutive, snowball |  | Purposive (intentionally selected).  The participants were group leaders in the pilot study and were selected to participate in the study following the pilot. |
| Method of approach | 11 | How were participants approached? e.g. face-to-face, telephone, mail, email |  | Researcher called the participants to schedule interview venue, day and time. During the interview day, participant consented, interviewed and reimbursed. |
| Sample size | 12 | How many participants were in the study? |  | 25 peer group leaders |
| Non-participation | 13 | How many people refused to participate or dropped out? Reasons? |  | None (100% participation on the qualitative portion), (however I will see how many participants complete the Redcap demographic survey when it is distributed) |
| *Setting* | | |  |  |
| Setting of data collection | 14 | Where was the data collected? e.g. home, clinic, workplace |  | 4 sites in Tanzania  Moshi – Majengo and KCMC Office  Mbeya – Psychologist Office  Ifakara – Chronic Disease Clinic of Ifakara (CDCI)  Mwanza – Baylor’s Office & Rock Beach |
| Presence of non-participants | 15 | Was anyone else present besides the participants and researchers? |  | No |
| Description of sample | 16 | What are the important characteristics of the sample? e.g. demographic data, date |  | All participants are living with HIV, mix of 13 males and 12 females, the age range (23-31) when in depth interviews were conducted. |
| *Data collection* | | |  |  |
| Interview guide | 17 | Were questions, prompts, guides provided by the authors? Was it pilot tested? |  | The interview guide was not pilot tested but interviewers were free to probe or follow-up as needed. (Not entirely sure)  The questions were created by a qualitative expert prior to distribution of the survey. |
| Repeat interviews | 18 | Were repeat interviews carried out? If yes, how many? |  | No |
| Audio/visual recording | 19 | Did the research use audio or visual recording to collect the data? |  | All interviews were audio recorded |
| Field notes | 20 | Were field notes made during and/or after the interview or focus group? |  | Field notes were made on the in depth interview document and transferred to Microsoft Excel for a debriefing form. |
| Duration | 21 | What was the duration of the interviews or focus group? |  | The average duration of the interviews was 74 minutes  Range:(60 minutes – 113 minutes) |
| Data saturation | 22 | Was data saturation discussed? |  | Yes |
| Transcripts returned | 23 | Were transcripts returned to participants for comment and/or |  | No |
| **Domain 3: analysis and findings** |  |  |  |  |
| *Data analysis* |  |  |  |  |
| Number of coders | 24 | How many data coders coded the data? |  | Two data coders coded a total of 8 transcripts with a subset of 2 transcripts from each of the four sites. One of those coders coded all 25 transcripts. |
| Description of coding tree | 25 | Did authors provide a description of the coding tree? |  | No, but the codes largely matched the interview guide questions and the guide was included as a supplemental document? |
| Derivation of themes | 26 | Were themes identified in advance or derived from the data? |  | Thematic domains were identified a priori (facilitators and barriers) but specific themes were derived from the data (Created after seeing the transcript responses) |
| Software | 27 | What software, if applicable, was used to manage the data? |  | Nvivo 12 and Microsoft Excel for rapid analysis |
| Participant checking | 28 | Did participants provide feedback on the findings? |  | Not yet, but this is a good idea? |
| *Reporting* |  |  |  |  |
| Quotations presented | 29 | Were participant quotations presented to illustrate the themes/findings? Was each quotation identified? e.g. participant number |  | I plan to do this.  I can use participant numbers as well. |
| Data and findings consistent | 30 | Was there consistency between the data presented and the findings? |  | Will answer this later. |
| Clarity of major themes | 31 | Were major themes clearly presented in the findings? |  | Will answer this later. |
| Clarity of minor themes | 32 | Is there a description of diverse cases or discussion of minor themes? |  | Will answer this later. |

From: Tong *et al.* *International Journal for Quality in Health Care*; (2007) 19;6:349-357.
