# Supplementary material for: Peer-led interventions: Exploring the peer group leader experience of delivering Sauti ya Vijana, a group-based mental health intervention for youth living with HIV in Tanzania
Source: PLOS Ment Health. 2026 Jan 21;3(1):e0000512. doi: 10.1371/journal.pmen.0000512 (PMC12822943; doi:10.1371/journal.pmen.0000512)
Supplement: Attachment — Submitted filename: Response to Reviewers.docx [file pmen.0000512.s006.docx]

**Manuscript PMEN-D-24-00520**

**Response to Reviewers**

Dear reviewers,

Thank you for taking the time to review and comment upon our manuscript “Peer-led interventions: exploring the peer group leader experience of delivering a group-based mental health intervention for youth living with HIV in Tanzania” for publication in *PLOS Mental Health*. We appreciate the time and effort that you and the reviewers dedicated to providing feedback on our manuscript and are grateful for the insightful comments on and valuable improvements to our paper. We have incorporated most of the suggestions made by the reviewers. Those changes are highlighted within the manuscript. Please see below, in blue, for a point-by-point response to the reviewers’ comments and concerns. All page numbers refer to the revised manuscript file without tracked changes.

Thank you again for your thoughtful comments.

Sincerely,

Chinenye Agina

**Journal Requirements:**

1. Please include a complete copy of PLOS’ questionnaire on inclusivity in global research in your revised manuscript. Our policy for research in this area aims to improve transparency in the reporting of research performed outside of researchers’ own country or community. The policy applies to researchers who have travelled to a different country to conduct research, research with Indigenous populations or their lands, and research on cultural artefacts. The questionnaire can also be requested at the journal’s discretion for any other submissions, even if these conditions are not met. Please find more information on the policy and a link to download a blank copy of the questionnaire here: https://journals.plos.org/plosmentalhealth/s/best-practices-in-research-reporting. Please upload a completed version of your questionnaire as Supporting Information when you resubmit your manuscript.

**Author response:** We’ve completed the PLOS’ questionnaire on inclusivity in global research and added a sentence underneath the Ethical Considerations subheading on page 11/line 221-223.

*“Additional information regarding the ethical, cultural, and scientific considerations*

*specific to inclusivity in global research is included in the Supporting Information (S3 File).”*

2. We have noticed that you have a list of Supporting Information legends in your manuscript. However, there are no corresponding files uploaded to the submission. Please upload them as separate files with the item type 'Supporting Information'.

**Author response:** Thank you! We’ve made sure to include the corresponding files in the submission. We’ve included the names below.

- S1 File: In-Depth Interview Question Guide
- S1 Table: COREQ COnsolidated criteria for REporting Qualitative research
- S2 File: Codebook for In-depth Interviews
- S2 Table: Description of 25 PGLs
- S3 File: Inclusivity in Global Research Questionnaire

3. In the online submission form, you indicated that your data will be submitted to a repository upon acceptance. We strongly recommend all authors deposit their data before acceptance, as the process can be lengthy and hold up publication timelines. Please note that, though access restrictions are acceptable now, your entire minimal dataset will need to be made freely accessible if your manuscript is accepted for publication. This policy applies to all data except where public deposition would breach compliance with the protocol approved by your research ethics board. If you are unable to adhere to our open data policy, please kindly revise your statement to explain your reasoning and we will seek the editor's input on an exemption.

**Author response:** Please see our revised statement: “All data in this study are derived from qualitative interviews. Every care has been taken to ensure anonymity of the data in the submitted manuscript, however, given the named regional location and positions of the study participants, freely available transcripts would jeopardize the conditions of our IRB ethical approvals. Deidentified data may be made available upon request from the senior (DD) after obtaining an appropriate data transfer agreement. Data is available upon request from Duke University after submission of a form found at: https://irb.duhs.duke.edu/about-us/contact-us. Please reference IRB #Pro00109309.”

4. Some material included in your submission may be copyrighted. According to PLOS’s copyright policy, authors who use figures or other material (e.g., graphics, clipart, maps) from another author or copyright holder must demonstrate or obtain permission to publish this material under the Creative Commons Attribution 4.0 International (CC BY 4.0) License used by PLOS journals. Please closely review the details of PLOS’s copyright requirements here: PLOS Licenses and Copyright. If you need to request permissions from a copyright holder, you may use PLOS's Copyright Content Permission form.

Please respond directly to this email or email the journal office and provide any known details concerning your material's license terms and permissions required for reuse, even if you have not yet obtained copyright permissions or are unsure of your material's copyright compatibility.

**Author response:** Thank you for the reminder! We’ve made sure to reach out to specific parties for any items with potential copyright issues.

Potential Copyright Issues:

5. Fig2.tif : Please confirm whether you drew the images / clip-art within the figure panels by hand. If you did not draw the images, please provide (a) a link to the source of the images or icons and their license / terms of use; or (b) written permission from the copyright holder to publish the images or icons under our CC-BY 4.0 license. Alternatively, you may replace the images with open source alternatives. See these open source resources you may use to replace images / clip-art: <https://commons.wikimedia.org> or <https://openclipart.org/>.

**Author response:** As suggested by the editor, we've received written permission from the copyright holder to use the Fig2.tif image in this paper. We’ve added local HIV prevalence rates for each study location by hand. We’ve shared the CC BY 4.0 License form with the journal office.

6. Figure 2: please (a) provide a direct link to the base layer of the map (i.e., the country or region border shape) and ensure this is also included in the figure legend; and (b) provide a link to the terms of use / license information for the base layer image or shapefile. We cannot publish proprietary or copyrighted maps (e.g. Google Maps, Mapquest) and the terms of use for your map base layer must be compatible with our CC-BY 4.0 license.

Note: if you created the map in a software program like R or ArcGIS, please locate and indicate the source of the basemap shapefile onto which data has been plotted.

**If your map was obtained from a copyrighted source please amend the figure so that the base map used is from an openly available source. A**lternatively, please provide explicit written permission from the copyright holder granting you the right to publish the material under our CC-BY 4.0 license.

Please note that the following CC BY licenses are compatible with PLOS license: CC BY 4.0, CC BY 2.0 and CC BY 3.0, meanwhile such licenses as CC BY-ND 3.0 and others are not compatible due to additional restrictions.

If you are unsure whether you can use a map or not, please do reach out and we will be able to help you. The following websites are good examples of where you can source open access or public domain maps:

* U.S. Geological Survey (USGS) - All maps are in the public domain. (http://www.usgs.gov)

* PlaniGlobe - All maps are published under a Creative Commons license so please cite “PlaniGlobe, http://www.planiglobe.com, CC BY 2.0” in the image credit after the caption. (http://www.planiglobe.com/?lang=enl)

* Natural Earth - All maps are public domain. (http://www.naturalearthdata.com/about/terms-of-use/)

**Author response:** As suggested by the editor, we've received written permission from the copyright holder to use the Fig2.tif image in this paper which includes the map of Tanzania. We’ve shared the CC BY 4.0 License form with the journal office.

**Reviewer #1**

This clearly written manuscript addresses the important topic of the perceptions of peer group leaders on their experiences delivering a mental health intervention in Tanzania. Given the recent increase of lay health worker-delivered mental health interventions in both LMIC and HIC settings, and particularly the focus on having these delivered by “peers” this is a timely and relevant focus. The authors conducted a qualitative sub-study comprised of in-depth interview data from the 25 Peer Group Leaders (PGLs) who delivered a mental health intervention for youth living with HIV in a trial conducted in Tanzania. There are some important findings about the motivators and challenges of working as a PGL, and some good implications for future programming. However, there are some areas that could benefit from further clarification and/or analysis. Although the study is stated to be based on the CFIR Implementation Science Framework, very few of the domains of this framework appear to have been addressed in the interviews and the Results are not presented in a way that shows how they relate to this framework. Further work is needed to specify and flesh-out sub-themes in the data, and to elucidated any sub-group differences. Specific comments and questions by section are given below.

**Author response:** Thank you for your feedback!

**Abstract:**

1. The authors should mention the number of PGL interviews that were conducted for the study in the abstract.

**Author response:** Thank you for the suggestion. We have updated the sentence to include the number of PGL interviews that were conducted for the study in the abstract on page 2/line 28-29.

*“In depth interviews (IDIs) (N=23) were conducted in 2023 with PGLs after delivering the scaled pilot test of SYV.”*

**Introduction:**

2. Line 46: The statement that 62.5% of mental disorders occur before age 25 needs clarification. Does this mean that most people with mental illness begin to experience their mental disorders before the age of 25, or something else?

**Author response:** Thank you for your comment. We’ve revised this language to reflect that 62.5% of individuals with a mental disorder first experience onset before the age of 25, based on findings from a large-scale meta-analysis on page 3/line 45-47.

*"Young adulthood is a significant developmental transition, and a large-scale meta-analysis*

*of 192 epidemiological studies found that 62.5% of individuals with a mental disorder experience onset before the age of 25 [4]."*

3. Lines 57-59: Is the first sentence presenting findings from a single local study or national data?

**Author response:** As suggested by the reviewer, we’ve revised the text to specify that this study is from a single local study on page 3-4/line 58-60.

“*The prevalence of depressive symptoms among Tanzanian adolescents living with HIV*

*(ages 7-18 years) was 27% in one local study conducted in a rural district of Tanzania,*

*compared to 6% among age-matched HIV-negative peers, using the Children Depression*

*Inventory II [13].*”

4. Line 63: What is the definition of “professional” in this sentence about the mental health workforce? Tanzania’s mental health workforce comprises of 1.31 4 63 professionals per 100,000 population, whereas in child and adolescent mental health services, this 64 number is 0.22 per 100,000 population [13].

**Author response:** Thank you for this comment. In this context, "mental health professionals" refers to psychiatrists, mental health nurses, psychologists, social workers, and other specialized mental health workers (e.g., occupational therapists), including both governmental and non-governmental personnel. We have clarified the sentence accordingly on page 4/line 64-65.

*“Tanzania’s mental health workforce, which includes psychologists, social*

*workers, and other specialized mental health workers, comprises 1.31 professionals per 100,000 population. In child and adolescent mental health services, this number drops to 0.22 per 100,000 population [15].”*

**Methods:**

5. Lines 98-99: The authors should specify whether SYV was developed originally for the study or is based/adapted on an evidence-based model implemented elsewhere. The multiple techniques being used (CBT, IPT, Motivational Interviewing) makes me wonder about the suitability for delivery by lay health workers with only a 2-week training.

**Author response:** As suggested by the reviewer, we have specified that SYV was adapted based on evidence-based models implemented elsewhere. Additional information can be found throughout the rest of the paragraph on page 5/line 98-103.

*“SYV is a peer-led, group-based mental health intervention for YLWH that aims to increase coping skills to manage common stressors and life challenges (Fig 1) [31]. It was co-designed in Tanzania with Tanzanian YLWH, a pediatric infectious diseases physician, and a clinical psychologist and incorporates components of evidence-based models to meet the needs YLWH described in our formative work [30].*

Lines 98-99: The authors should also specify here the age range of the YLWH included in the SYV trial.

**Author response:** As suggested by the reviewer, we’ve included the age range of the YLWH included in the SYV trial on page 5/line 102-103.

*“SYV was co-created in 2016 and was evaluated in a pilot study in Kilimanjaro for youth 12-24*

*years of age [19]..”*

6. Lines 179-187: If this study was really informed by the CFIR framework, why were more of the CFIR domains not investigated in the interviews and analysis?

**Author response:** Thank you for the question. The overall parent study was informed by CFIR (clinical trial, implementation research outcomes, and qualitative data), but our current study for this manuscript and the associated interview guides only cover data from two CFIR domains, which is by design (inner setting and individuals involved). We only mentioned CFIR in the methods section to aid in additional context for study. It was not our intention to analyze data specifically by CFIR domain or characteristic as they do not align with our more targeted research questions. Other types of stakeholders contributed data to other CFIR domains but that is outside the scope of this study for reporting. We have added new text in the “In-depth Interviews” methods section that explains more clearly for the reader how CFIR fits in our overall study design and the current study on page 9-10/line 183-194.

*“The overall parent study [33] is a hybrid implementation-clinical trial (ClinicalTrials.gov #NCT05374109) with a complementary qualitative study design informed by the Consolidated Framework for Implementation Research (CFIR) to ensure data collection from a variety of stakeholder perspectives. The in-depth interview (IDI) guides for this current study are tailored in order to explore and understand the role of PGLs in the implementation of the SYV mental health intervention* ***[35]****. The IDI guide was created in accordance with the current study objectives of understanding the group leaders’ motivation, experience, and identifying ways to improve SYV in the future. Most of the IDI guide questions thus fell under the CFIR domains of ‘individuals involved’ and ‘inner setting’. Our thematic results are organized under these two domains but there is also a larger heading of intervention sustainability because this cuts across both CFIR domains and better aligns with our IDI guide. The topics under these domains included issues such as identity, motivations, experience as a group leader, group leader training, impact of intervention on PGLs, sustainability, and recommendations. The IDI guide included 32 open-ended questions (S1 File).”*

7. Lines 197-200: Was the Tanzanian qualitative researcher involved in the full coding and analysis, or just this double-coding of 8 transcripts? This is not clear.

**Author response:** Thank you for your question. We’ve revised this language on page 10/line 204-207.

*“To ensure the qualitative analysis was contextually grounded, a second Tanzanian*

*qualitative researcher (FN) double-coded eight transcripts (~25% of all transcripts), two*

*transcripts from each site, aiming to identity a similar interpretation of codes (34). No additional codes were identified during this process [38].”*

8. Line 209: I am noting that COREQ includes criteria for reporting qualitative research not for conducting it.

**Author response:** Thank you for this wording clarification. We’ve revised this language on page 11/line 216-217.

*“This study reports further details via the Consolidated Criteria for Reporting Qualitative*

*Research (COREQ) (see S1 Table) [44].”*

**Results:**

9. Overall: It would be better to organize the Results according to your CFIR conceptual framework, instead of just the classic and simplified Motivators and Challenges themes. More sub-themes are also needed to more clearly flesh out the themes. Disparate sub-themes seem to be lumped together in the results. The whole analysis would be improved by fleshing out and specifying sub-themes.

**Author response:** Thank you for this comment. Upon reflection and in recognition that our goal was not to analyze data strictly by CFIR domains and constructs, we have significantly reorganized the results section without adding new analyses so that the overarching headers align with the 2 CFIR domains (Individuals Involved & Inner Setting) and plus a cross-cutting header about sustainability. We have also revised many of our theme titles to be more descriptive/summative of the actual identified theme (i.e. “Collaborative relationships with colleagues” is now “SYV experience helped foster collaborative relationships between PGL colleagues.” We believe this new flow will greatly aid the reader through our results section. Thank you for the suggestion. Below is an outline of the newly organized results section:

- Description of PGLs
- Themes under the “Individuals Involved” CFIR Domain
  - *Desire to help youth as motivation for PGL role*
  - *Increased confidence as a perceived benefit*
  - *Recognition of shared personal benefit with youth*
  - *Potential challenge of SYV Sessions is the Emotional Impact for PGLs*
  - *PGLs experienced mixed community perceptions about their role*
- Themes under the “Inner Setting” CFIR Domain
  - *SYV experience helped foster collaborative relationships between PGL colleagues*
  - *Social Dynamics between PGLs could also be challenging*
  - *Compensation concerns were common*
  - *Limitations of PGL role*
  - *Challenges of teaching competence and cross-site PGL support and supervision*
- Themes under Sustainability of SYV intervention (this had a mix of individuals and setting so it is kept as is and explained in methods)
  - *Intervention Goals and Expansion*
  - *Career advancement*
  - *Feedback and Recommendations*

10. Line 248: Applying skills learned to their own daily lives, for example, seems like a separate theme than “confidence”.

**Author response:** Thank you for pointing this out. We’ve revised the theme to be “increased confidence as a perceived benefit.” You can see this change on page 12/line 252.

11. Lines 291-294: It is not clear from this quote how this effect stems from relationships with colleagues (the theme of this section).

**Author response:** Thank you for pointing this out. We’ve moved this quote under Themes under the “Inner Setting” CFIR Domain and placed it under the “SYV experience helped foster collaborative relationships between PGL colleagues.” You can find the new placement on page 17/line 343-346. The quote now explains how the role helped the PGL foster relationships between their colleagues.

12. Line 343: The theme “inclusivity and collaboration” is included in the Challenges section (I believe), but is this a challenge? Should this be labeled “lack of inclusivity and collaboration”?

**Author response:** We’ve moved this under the Themes under the “Inner Setting” CFIR domain and renamed the subtheme to be “Challenges of teaching competence and cross-site PGL support and supervision.” You can find the new placement on page 20/line 381-382.

13. Lines 371-37: This information on increased respect and recognition within the community seems that it should be moved up with the benefits and facilitators for the PGLs individually.

**Author response:** Thank you for your suggestion. We’ve moved this information under the larger Theme “Theme under the “Individuals Involved” CFIR Domain specifically under the subtheme “PGLs experienced mixed community perceptions about their role.” You can find the new placement on page 15/line 308.

14. Lines 379-389: It is not clear why teaching competence and supervision are lumped together. These seem like separate sub-themes.

**Author response:** We’ve moved the information under the Teaching Competence and Supervision theme under the Themes under the “Inner Setting” CFIR domain, specifically the subtheme “Challenges of teaching competence and cross-site PGL support and supervision.” You can find the new placement on page 18/line 381.

15. Line 408: This theme about career advancement seems that it would be better placed above as it is a concern of the PGLs about themselves and their future.

**Author response:** While we appreciate the reviewer’s feedback, we respectfully disagree. We think the subtheme about career advancement makes a valuable contribution to the larger theme regarding the sustainability of the intervention. We’ve gotten rid of the “Challenges faced in the PGL role” theme that was in the first version of our paper.

16. Were there any sub-group differences in the data, for example male vs. female PGLs? These should be analyzed and results presented.

**Author response:** Thank you for pointing this out. Although we agree that this is an important consideration to include in the future, it is beyond the scope in this manuscript because male and female PGLs experienced the same benefits and challenges based on their transcript. The study focused on having an equal amount of male and female PGLs and no significant differences were reported during the analysis phase of the transcripts.

**Discussion:**

17. Lines 459-467: It is not clear how the citations in this paragraph relate to the statements made here that seem to be about the results of the current study, not from the literature.

**Author response:** We agree with the reviewer’s assessment. Accordingly, throughout this section, we have revised this paragraph to clearly distinguish between our findings and the literature on page 23/line 469-474.

*“The exploration of motivators for the PGL role in this study revealed a range of various*

*experiences and values that drove their engagement in the role. A strong desire to positively impact the lives of youth consistently emerged as a key motivator. For example, one PGL shared how the experience of losing peers motivated them to apply for the role, emphasizing how personal loss can shape career choices. Several PGLs also described a sense of responsibility not only to the youth in the intervention but also to peers in their broader community.*

*These findings align with previous studies showing that holding roles with meaningful impact on youth can be deeply fulfilling for peer leaders [46], and that personal connections to the role often influence motivation and commitment [22,47]. In addition, prior research has found that shared experiences with the target population can enhance facilitators’ ability to relate to participants and foster engagement, which we also observed in our study [41,48].*

18. Lines 537-538: How will this recommendation affect cost and sustainability of this intervention in health systems?

**Author response:** Thank you for this comment. We’ve made sure to expand on how having local supervisors presenting may affect the cost and sustainability of SYV in health systems on page 26/line 553-557.

*“Two PGLs suggested that having local supervisors present in person for supervision would*

*be more valuable. However, this approach would increase costs and could affect*

*sustainability, as supervisors currently have primary clinical roles and supervision is*

*conducted in a hybrid format to accommodate staffing and site constraints.”*

**Tables and figures:**

19. The second column of the supplementary codebook file does not seem to add much as it is mainly just a repetition of the in-depth interview guide.

**Author response:** While we appreciate the reviewer’s feedback, we respectfully disagree. We think the second column of the supplementary file adds to the paper by chunking the in depth interview questions into different nodes that we used when analyzing the transcript.

**Reviewer 2**

This is an important paper with useful lessons for providers of similar interventions. The role of PGL clearly has a substantial effect on many of those interviewed.

**Author response:** Thank you!

1. In reference 1 (The UNAIDS 2022 data report) I couldn’t find the specific information cited here: 150,000 YLWH in Tanzania, 100,000 young women living with HIV. The paper says “This disparity is evident in HIV prevalence rates, with adolescent young women having a rate of 1.7, nearly twice the rate of adolescent young men at 0.9”. I don’t know what this means. A prevalence is not a rate, and 1.7 is neither a rate or a prevalence, it’s just a number. What is it you want to say? What occurs at a rate of 1.7 what, per what?

**Author response:** Thank you for pointing this out. We’ve replaced the number of YLWH in Tanzania with the estimated number of new HIV infections among youth in Tanzania. The prevalence represents the proportion of youth that were living with HIV in Tanzania during a specific time period. You can find these changes on page 3/line 52-56.

*“In 2023, there were an estimated 150,000 youth (15-24 years of age) living with HIV and a staggering19,000 new HIV infections [11]. A gender disparity is evident with nearly two fold higher prevalence among adolescent girls and young women living with HIV (1.3% ) compared to adolescent boys and young men (0.6%) [12].*

2. I’m not clear about the relevance of the information in the introduction that youth who found out their HIV status independently had poorer ART adherence. All the youth in this study already know their HIV status. However, if this point is relevant, you might be interested in the paper below which found that youth in Zimbabwe with symptoms of anxiety/depression who had not told anyone their HIV status had poorer viral suppression (and also that many youth knew their status although their parents did not think they they knew it).
   Simms et al (2021) Risk factors for HIV virological non-suppression among adolescents with common mental disorder symptoms in Zimbabwe: a cross-sectional study. JIAS <https://doi.org/10.1002/jia2.25773>

**Author response:** While we appreciate the reviewer’s feedback, we respectfully disagree. The cited articles were included to illustrate broader challenges that YLWH face globally, with medication adherence being one of them. This sentence is intended to provide context for the complex realities YLWH navigate, even after learning their HIV status. We believe this framing adds important background for readers and helps situate the relevance of the intervention. To clarify, this paragraph is not focused specifically on SYV participants, but rather on the wider challenges faced by YLWH.

3. It is good to learn that PGLs have benefitted in confidence, friendships, mental health and self-acceptance from their work. However, the discussion should address whether this means that perhaps at the start of the program, some of them should not have been supervising groups of adolescents. Despite the name, the PGLs are not peers of the youth. They are adults who are taking responsibility for youth in the groups. It is definitely important to provide support systems for the PGLs to manage emotional discomfort caused by the role, as you said in the discussion.

**Author response:** Thank you for your feedback. We've expanded on the role of PGLs as adults and clarified the unique position they hold, given that many were previously youth at the same clinics or served as peer educators. We have also addressed the concern regarding whether all PGLs were ready to supervise adolescents at the start of the program by emphasizing the importance of screening, training, and ongoing supervision to support professional boundaries and preparedness on page 24/line 514-518.

*“While PGLs are adults who carry responsibility for youth, many entered the program through engagement with the clinics, often beginning as adolescent patients or peer educators. This background allows them to relate to participants in a meaningful way, but it also highlights the importance of careful screening and structured support to ensure they are fully prepared for the supervisory role from the outset.”*

4. The section about social dynamics between PGLs is unclear. The quote on lines 326-328 doesn’t seem to be about issues being escalated, but the text says that it is.

**Author response:** Thank you for your feedback. We've replaced the quote with one that highlights issues being escalated between PGLs. You can find the revised text on page 17/line 353-355.

*“It’s the gossiping that I hate—when someone takes things to higher-ups before we’ve even agreed to share it. It was supposed to stay between us. Small issues, and yet someone’s already told the boss.” (Female PGL)*

**Minor comments**

1. What is Duke Box?

**Author response:** Thank you for pointing this out. We have explained what Duke Box is in the methods section on page 9/line 176-178.

*“A handheld audio recording device was used to record, and the de-identified recorded*

*interviews were uploaded to Duke Box, a HIPAA-compliant collaborative cloud storage*

*platform used by Duke University.”*

2. I recommend writing dates in full, eg. 25 October 2021 instead of 25/10/2021.

**Author response:** Thank you for your suggestion. All dates in the manuscript have been consistently written in full using the “Month Year” format (e.g., “November 2023”), including in the Methods section.

3. The abstract says the PGLs were aged 23-29 but the text says they were aged 23-31.

**Author response:** Thank you for pointing this out. The reviewer is correct, and we have changed the language of the abstract to specifically call out that PGLs were 23-29 at the time of hire.

The revised text reads as follows on page 2/line 26-27:

“*Peer group leaders (PGLs) aged 23 to 29 years at the time of hire and living with HIV*

*were trained to deliver SYV”*
