# Supplementary material for: Peer-led interventions: Exploring the peer group leader experience of delivering Sauti ya Vijana, a group-based mental health intervention for youth living with HIV in Tanzania
Source: PLOS Ment Health. 2026 Jan 21;3(1):e0000512. doi: 10.1371/journal.pmen.0000512 (PMC12822943; doi:10.1371/journal.pmen.0000512)
Supplement: Attachment — Submitted filename: 11.3 PLOS Response to Reviewers PMEN-D-24-00520.docx [file pmen.0000512.s007.docx]

**Manuscript PMEN-D-24-00520**

**Response to Reviewers**

Dear reviewers,

Thank you for taking the time to review and comment upon our manuscript “Peer-led interventions: exploring the peer group leader experience of delivering a group-based mental health intervention for youth living with HIV in Tanzania” for publication in *PLOS Mental Health*. We appreciate the time and effort that you and the reviewers dedicated to providing feedback on our manuscript and are grateful for the insightful comments on and valuable improvements to our paper. We incorporated all but one suggestion. We opted not to implement the recommendation to add an Outer Domain as described below. All changes are highlighted within the manuscript. Please see below, in blue, for a point-by-point response to the reviewers’ comments and concerns. All page numbers refer to the revised manuscript file with tracked changes.

Thank you again for your thoughtful comments.

Sincerely,

Chinenye Agina

**Journal Requirements:**

If the reviewer comments include a recommendation to cite specific previously published works, please review and evaluate these publications to determine whether they are relevant and should be cited. There is no requirement to cite these works unless the editor has indicated otherwise.

Please review your reference list to ensure that it is complete and correct. If you have cited papers that have been retracted, please include the rationale for doing so in the manuscript text, or remove these references and replace them with relevant current references. Any changes to the reference list should be mentioned in the rebuttal letter that accompanies your revised manuscript. If you need to cite a retracted article, indicate the article’s retracted status in the References list and also include a citation and full reference for the retraction notice.

Additional Editor Comments (if provided):

Address the comments raised by the reviewers


[Note: HTML markup is below. Please do not edit.]

Reviewers' comments:

Reviewer's Responses to Questions

**Comments to the Author**

1. If the authors have adequately addressed your comments raised in a previous round of review and you feel that this manuscript is now acceptable for publication, you may indicate that here to bypass the “Comments to the Author” section, enter your conflict of interest statement in the “Confidential to Editor” section, and submit your "Accept" recommendation.

Reviewer #1: (No Response)

Reviewer #2: All comments have been addressed

2. Does this manuscript meet PLOS Mental Health’s [publication criteria](https://track.editorialmanager.com/CL0/https:%2F%2Fjournals.plos.org%2Fmentalhealth%2Fs%2Fcriteria-for-publication/1/010f0199c2714f08-43279785-933f-41e1-afd9-f08bd566b807-000000/0SyBz-97S1UEiO-mAqfSP4L-auKjWMxJS3I7YygLZKE=231)? Is the manuscript technically sound, and do the data support the conclusions? The manuscript must describe methodologically and ethically rigorous research with conclusions that are appropriately drawn based on the data presented.

Reviewer #1: Yes

Reviewer #2: Yes

3. Has the statistical analysis been performed appropriately and rigorously?

Reviewer #1: N/A

Reviewer #2: Yes

4. Have the authors made all data underlying the findings in their manuscript fully available (please refer to the Data Availability Statement at the start of the manuscript PDF file)?

The [PLOS Data policy](https://track.editorialmanager.com/CL0/https:%2F%2Fjournals.plos.org%2Fmentalhealth%2Fs%2Fdata-availability/1/010f0199c2714f08-43279785-933f-41e1-afd9-f08bd566b807-000000/59Jh5gZucHfVUVWxQ29--M4cpvwd9Hm2ZmGZ4sZCm5M=231) requires authors to make all data underlying the findings described in their manuscript fully available without restriction, with rare exception. The data should be provided as part of the manuscript or its supporting information, or deposited to a public repository. For example, in addition to summary statistics, the data points behind means, medians and variance measures should be available. If there are restrictions on publicly sharing data—e.g. participant privacy or use of data from a third party—those must be specified.

Reviewer #1: Yes

Reviewer #2: No

**Author response:** Please see the revised statement that we submitted during our first round of revisions:

*“All data in this study are derived from qualitative interviews. Every care has been taken to ensure anonymity of the data in the submitted manuscript, however, given the named regional location and positions of the study participants, freely available transcripts would jeopardize the conditions of our IRB ethical approvals. Deidentified data may be made available upon request from the senior (DD) after obtaining an appropriate data transfer agreement. Data is available upon request from Duke University after submission of a form found at: https://irb.duhs.duke.edu/about-us/contact-us. Please reference IRB #Pro00109309.”*

5. Is the manuscript presented in an intelligible fashion and written in standard English?

PLOS Mental Health does not copyedit accepted manuscripts, so the language in submitted articles must be clear, correct, and unambiguous. Any typographical or grammatical errors should be corrected at revision, so please note any specific errors here.

Reviewer #1: Yes

Reviewer #2: Yes

6. Review Comments to the Author

Please use the space provided to explain your answers to the questions above. You may also include additional comments for the author, including concerns about dual publication, research ethics, or publication ethics. (Please upload your review as an attachment if it exceeds 20,000 characters)

**Reviewer #1**

The authors have generally done a very good job responding to the editor and reviewer comments and making relevant changes in the manuscript. The additional clarity on how CFIR was used in the qualitative data collection, analysis, and reporting of results is appreciated. There are a few remaining minor concerns:

- The response letter (response to Reviewer #1, response 1) indicates an N of 23 interviews, whereas elsewhere an N of 25 is indicated.

**Author response:** Thank you for mentioning this. I’m confirming that our original response #1 for Reviewer #1 included the language below in the abstract on page 2/line 28. This may have been confused with the age range in the abstract, which states that PGLs were aged 23 to 29 years at the time of hire.

*“25 In depth interviews (IDIs) were conducted in 2023 with PGLs after delivering the scaled pilot test of SYV.”*

- The abstract should be edited to better reflect the new thematic organization of results around the CFIR Individuals Involved and Inner Setting themes. The abstract still reflects the previous organization around benefits and challenges.

**Author response:** Thank you for mentioning this. I’ve updated the abstract to reflect the new thematic organization of the results around the CFIR Individuals Involved and Inner Setting, and themes on page 2/line 31-37.

*“Youth living with HIV (YLWH) face mental health challenges which negatively*

*influence their adherence to antiretroviral medication and HIV outcomes. In sub-Saharan Africa, where the majority of YLWH reside, there are few mental health professionals. Task-shifting interventions to lay peer leaders may be an effective strategy for addressing mental health challenges. This study aims to understand and evaluate peer group leaders’ experiences delivering a peer-led, group-based mental health intervention called The Voice of Youth (Sauti ya Vijana [SYV] in Swahili) to YLWH in Tanzania. Peer group leaders (PGLs) aged 23 to 29 years at the time of hire and living with HIV were trained to deliver SYV. The study took place across four regions in Tanzania. In depth interviews (IDIs) (N=25) were conducted in 2023 with PGLs after delivering the scaled pilot test of SYV. IDIs were audio-recorded in Swahili and translated and transcribed into English. Thematic analysis was applied using NVivo for coding and Excel to further summarize data and identify themes.* ***PGLs experiences are organized via according to two CFIR domains: Individuals Involved and Inner Setting. Under the Individuals Involved domain, PGLs described motivations such as a desire to help youth, increased confidence, and shared personal growth, as well as emotional challenges related to youth trauma. Within the Inner Setting domain, PGLs highlighted collaboration and collegial support alongside challenges related to social dynamics, compensation, and supervision. . Across themes, PGLs emphasized sustainability, offering recommendations to strengthen program expansion and long-term impact.*** *Insights from the PGLs can help enhance and position SYV for sustainability as Tanzania navigates scaling mental health care YLWH and also inform other peer-led mental health interventions in low-resource contexts.”*

- I would suggest that the information provided in the response letter on how the data were interrogated for sub-group differences and the lack of sub-group differences found in the data (response to Reviewer #1, response 16) should be included in the Methods section of the manuscript (Data analysis).

**Author response:** Thank you for recommending this. I’ve included this language in the Methods section of the manuscript in the data analysis section on page 12/line 221-224.

*“In our thematic analysis, we found no sub-group differences based on gender.”*

- One could argue that the sub-theme around community perceptions of the PGLs would fall under the Outer Setting domain of CFIR. The authors may want to consider separating this out in that way, along with any other Outer Setting findings.

**Author response:** We appreciate this comment. Our team reviewed the results carefully and while we see your point, the analysis highlights GLs perspectives. The community perceptions are from the perspective of the PGL and not related to interviewing the community directly (which we agree, would be more clearly outer setting). Given the findings relate to the PGL themselves (their individual experiences) we think this component of the PGL experience is best found in the individuals involved domain.

**Reviewer #2**

Thank you, all my comments have been addressed.

**Author response:** Thank you for reviewing.

- One very minor point - the prevalence of HIV in girls and young women (1.3%) is more than twice the prevalence in boys and young men (0.6%), not almost twice.

**Author response:** Thank you for pointing this out. We’ve revised this language in the Introduction section on page 4/line 60.

*“A gender disparity is evident, with more than twice the prevalence among adolescent*

*girls and young women living with HIV (1.3% ) compared to adolescent boys and young*

*men (0.6%) [12].”*
